# Handedness and the X chromosome: The role of androgen receptor CAG-repeat length

**DOI:** 10.1038/srep08325

**Published:** 2015-02-09

**Authors:** Larissa Arning, Sebastian Ocklenburg, Stefanie Schulz, Vanessa Ness, Wanda M. Gerding, Jan G. Hengstler, Michael Falkenstein, Jörg T. Epplen, Onur Güntürkün, Christian Beste

**Affiliations:** 1 Department of Human Genetics, Ruhr-University, 44780 Bochum, Germany; 2 Institute of Cognitive Neuroscience, Biopsychology, Department of Psychology, Ruhr-University, 44780 Bochum, Germany; 3 Leibniz Research Centre for Working Environment and Human Factors (IfADo), 44139 Dortmund, Germany; 4 Cognitive Neurophysiology, Department of Child and Adolescent Psychiatry, Faculty of Medicine of the TU Dresden, 01309 Dresden, Germany

## Abstract

Prenatal androgen exposure has been suggested to be one of the factors influencing handedness, making the androgen receptor gene (AR) a likely candidate gene for individual differences in handedness. Here, we examined the relationship between the length of the CAG-repeat in AR and different handedness phenotypes in a sample of healthy adults of both sexes (n = 1057). Since AR is located on the X chromosome, statistical analyses in women heterozygous for CAG-repeat lengths are complicated by X chromosome inactivation. We thus analyzed a sample of women that were homozygous for the CAG-repeat length (n = 77). Mixed-handedness in men was significantly associated with longer CAG-repeat blocks and women homozygous for longer CAG-repeats showed a tendency for stronger left-handedness. These results suggest that handedness in both sexes is associated with the AR CAG-repeat length, with longer repeats being related to a higher incidence of non-right-handedness. Since longer CAG-repeat blocks have been linked to less efficient AR function, these results implicate that differences in AR signaling in the developing brain might be one of the factors that determine individual differences in brain lateralization.

With more than 90% of all humans preferring to use their right hand, handedness is the most noticeable functional expression of cerebral lateralization in humans^1^. However, the precise molecular mechanisms that regulate handedness and other related forms of cerebral lateralization such as language dominance remain elusive^2^. Handedness is a complex, heritable trait, for which polygenic inheritance is assumed^3^, meaning that a large number of genetic factors with a small additive effect contribute to trait variance. Additive genetic effects were shown to account for about a quarter of the variance, with the remainder accounted for by non-shared environmental influences^4^,^5^,^6^,^7^. To date, linkage analyses and candidate gene studies have implicated a number of chromosome regions (2p12-q11, 10q26 and 12q21-23) and few specific genes (*LRRTM1*, *PCSK6* and *AR*) as influencing handedness^8^,^9^,^10^,^11^,^12^,^13^,^14^,^15^,^16^,^17^. Particularly interesting are the associations between the human androgen receptor (*AR*) gene and different aspects of handedness, since the interrelationships constitute a conceptual bridge between the theories that invoke testosterone as a factor in the development of cerebral asymmetries^11^,^16^,^18^,^19^,^20^ with theories proposing that the X chromosome contains a locus that influences the direction of hand preference^21^,^22^. A higher prevalence of left-handedness among men appears to be a robust finding^23^,^24^, and a large number of studies reporting sex differences in brain structure and function underlying language processes have been published^25^,^26^,^27^.

Since prenatal testosterone (pT) has organizing effects on brain and behavior, and male fetuses temporarily produce more than 2.5 times the levels observed in females^28^, various theories and hypotheses about the role of pT in the development of brain lateralization have been developed to explain these sex differences: Geschwind and Galaburda (1985), hypothesized that high levels of pT during early development slow down the development of parts of the left hemisphere, predicting that higher pT concentrations are related to a decrease of language lateralization to the left hemisphere and increased incidence of left-handedness^18^. Witelson & Nowakowski (1991), on the other hand, formulated the callosal hypothesis which is based on the finding that non-consistent right-handed males have larger callosal areas than consistent right-handed males. In this context consistent handedness means that the preferred hand is consistently the left or the right over all items in a handedness questionnaire^20^. The hypothesis implies that pT enhances axonal pruning in the corpus callosum, predicting that via the resulting reduction of inter-hemispheric transfer of information the degree of lateralization will increase^19^,^20^.

The human *AR* gene is located on chromosome Xq11.2–q12 and codes for a nuclear transcription factor that mediates the actions of testosterone and dihydrotestosterone^29^. The gene comprises a highly polymorphic (CAG)_n_ repeat in exon 1 encoding a glutamine tract in the N-terminal transactivation domain of the protein^30^. A negative linear association between AR function and the CAG-repeat block size is generally assumed, although controversial data exist as well^31^,^32^.

Interestingly, a relationship of *AR* CAG-repeat length and handedness has been suggested by two previous studies. Medland et al. (2005) examined the role of the *AR* CAG-repeat length in association with handedness for writing in 783 twin pairs^11^. They found that females with longer CAG-repeats were more likely to be left-handed, while the opposite effect was observed for males, where longer CAG-repeats correlated with a lower incidence of left-handedness. Under the assumption that the length of the *AR* CAG-repeat is positively correlated with testosterone effects in males, and negatively correlated in females, they conclude, consistent with the callosal hypothesis, that the likelihood of left handedness is increased in those individuals with variants of the *AR* associated with lower testosterone levels. Recently, the association with the *AR* CAG-repeat and handedness was confirmed in an independent sample of 180 males by Hampson and Sankar (2012)^16^. However, the direction of the effect was different from that observed previously. Here, mixed-handedness, as assessed by inventory-derived laterality scores, was significantly associated with longer CAG-repeats in males. Besides this inconsistency between the two studies, that might have been caused by methodological differences in phenotype assessment and/or sample size, statistical analyses in women heterozygous for CAG-repeat lengths is complicated by X chromosome inactivation. In order to address this issue and make an unbiased investigation of the molecular determinants of sex differences in human handedness, we performed an association study of *AR* CAG-repeat lengths and different handedness phenotypes. Overall, we tested in a sample of 1057 unrelated healthy Caucasian adults and specifically analyzed handedness in a subsample of women that were homozygous for CAG-repeat length (n = 77).

## Results

### Handedness

Table 1 shows the average values for all dependent variables for men, women and the overall sample. There were no significant sex differences when comparing left *vs.* right-handedness (direction 1, χ^2^ = 0.37; p = 0.54; odds ratio: 1.15) or consistent *vs.* inconsistent handedness (direction 2, χ^2^ = 0.86; p = 0.35). Although also not significant, the laterality quotient (LQ) was slightly higher in men as compared to women (73.84 *vs.* 75.59, t_(1054)_ = 0.61; p = 0.55). Because the *AR* gene is X-linked, males are hemizygous for the CAG-repeat polymorphism (only one allele). In contrast, females carry two alleles, of which only one is expressed in any cell because of the phenomenon of X chromosome inactivation. Therefore, in accordance with previous studies^11^, male and female data were analyzed separately.

### Data in males

**Figure 1:**
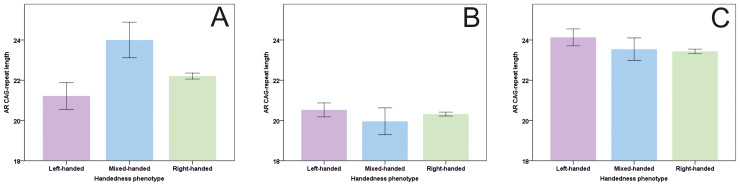
Handedness Direction 2 phenotypes (left-handed, mixed-handed, right-handed) in relation to CAG-repeat length for male (A) and female participants (B: short allele; C: long allele). Error bars show standard error.

The *AR* CAG-repeats in males ranged from 6 to 35, with a mean repeat length of 22.20 (+/−3.00), a finding that is in line with previous data on Caucasian populations^33^. Similar to the analysis performed by Hampson and Sankar (2012)^16^, the association between CAG-repeat length and Direction 2 was investigated using a univariate ANOVA with CAG-repeat length as dependent variable and handedness phenotype (LH, MH, RH) as fixed factor. For Direction 1 and Consistency, unpaired t-tests with handedness phenotype (Direction 1: LH, RH; Consistency: Inconsistent, Consistent) as grouping factor and CAG-repeat length as dependent variable were calculated to test the association between these variables and CAG-repeat length. While no significant effect was observed for Direction 1 (t_(428)_ = 1.15; p = 0.25), the results for Direction 2 (F_(2, 427)_ = 4.13; p = 0.017; MS_Error_ = 8.83; partial η^2^ = 0.02) indicated that mixed-handed males (24.00+/−0.82) had a significantly higher CAG-repeat length than left-handed (21.22+/−0.53) or right-handed males (22.21+/−0.15) (Fig. 1). For consistency, the effect of handedness phenotype also failed to reach significance (t_(428)_ = 1.53; p = 0.13).

**Figure 2:**
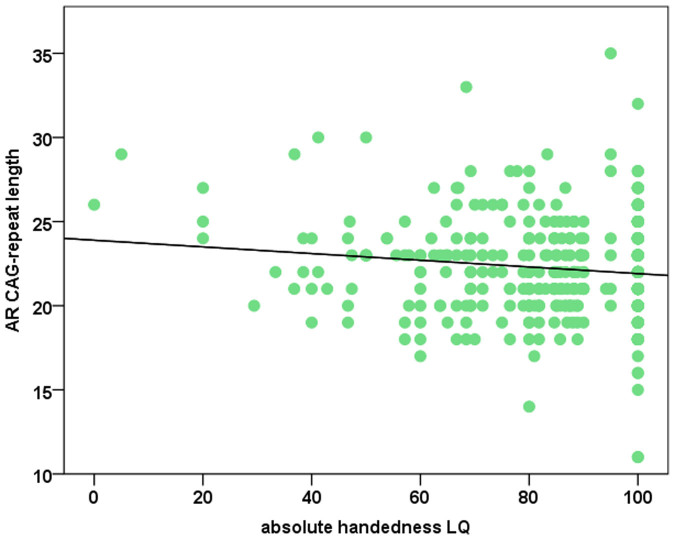
Absolute handedness LQ in relation to the *AR* CAG-repeat length. The black line indicates the central tendency of the relationship between the two variables.

To investigate, whether the relation between mixed handedness (as reflected by low absolute LQ numbers) and CAG-repeat length was not only qualitative but also quantitative, we calculated a two-sided Neyman-Pearson correlation coefficient between absolute LQ and CAG-repeat length. Interestingly, men with lower absolute LQ had significantly higher CAG repeat lengths (r = −0.12; p = 0.01; polyserial correlation coefficient: −0.12; Fig. 2). Finally, to investigate, whether a medium length CAG-repeat might lead to an increase in functional activity as compared to the long or short CAG-repeat blocks as recently proposed by Nenonen et al., we compared participants with average CAG-repeat lengths (21–23 CAGs) with the rest of the cohort harboring longer or shorter repeats^32^. An independent samples t-test did not reveal any significant differences between the two groups regarding LQ (t_(428)_ = 0.71; p = 0.48).

### Data in females

The number of *AR* CAG repeats in females ranged from 8 to 35 with a mean length of 20.32 (+/−2.34) for the short allele and 23.48 (+/−2.63) for the long allele, a finding that is in line with previous data on Caucasian populations^33^. As with the data in males, the association between CAG-repeat length and handedness was investigated using a univariate ANOVA for Direction 2 and unpaired t-tests for Direction 1 and Consistency. For the short allele, all effects failed to reach significance in female participants (Direction 1: t_(624)_ = −0.55; p = 0.58; Direction 2: F_(2, 623)_ = 0.40; p = 0.65; MS_Error_ = 5.48; Consistency: t_(624)_ = −0.44; p = 0.66). For the long allele, Direction 1 (t_(624)_ = −1.16; p = 0.25) and Direction 2 (F_(2, 623)_ = 1.26; p = 0.29; MS_Error_ = 6.92) also failed to reach significance, while for Consistency a trend (t_(624)_ = 1.87; p = 0.06) towards a higher repeat length in inconsistently handed females (23.68+/+2.66) as compared to consistently handed females (23.28+/+2.48) was obtained. For females, the correlation between absolute LQ and CAG-repeat length failed to reach significance for both the short (r = 0.04; p = 0.31; polyserial correlation coeffient: 0.04) and the long alleles (r = −0.05; p = 0.19; polyserial correlation coeffient: −0.05).

**Figure 3:**
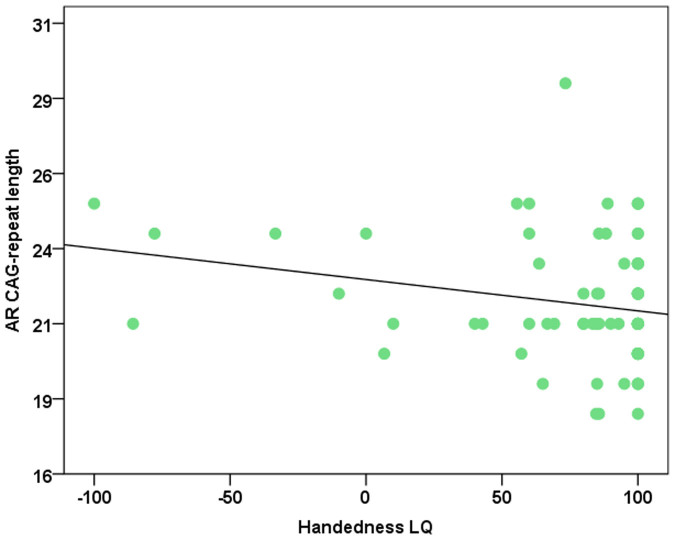
Handedness LQ in relation to the *AR* CAG-repeat length for the 77 female participants with homozygous CAG-repeat genotypes. The black line indicates the central tendency of the relationship between the two variables.

Additionally, to investigate the influence of CAG-repeat length in females independent of X inactivation, a separate analysis of the 77 female participants with homozygous CAG-repeat genotypes was conducted. For Direction 1 (t_(75)_ = −1.76; p = 0.08), Direction 2 (F_(2, 74)_ = 1.17; p = 0.31; MS_Error_ = 4.01) and Consistency (t_(75)_ = 0.79; p = 0.43) the effect failed to reach significance. While no significant correlation between CAG-repeat length and absolute LQ was observed (r = −0.11; p = 0.31; polyserial correlation coeffient: −0.11), a strong trend was recognizable for a correlation between CAG-repeat length and LQ (r = −0.22; p = 0.05; polyserial correlation coeffient: r:−0.22), indicating that in homozygous women longer CAG-repeats had a tendency to be related with stronger left-handedness (Fig. 3).

## Discussion

Handedness is a sex-dependent phenotype, with males showing a higher frequency of left-handedness than females^27^,^34^,^35^. Due to this sex difference, different hypotheses have been put forward that suggest an important role of androgens, specifically testosterone, for the development of handedness. Here, we present data showing that mixed-handed men differ from strongly right- and left-handed men in their *AR* CAG-lengths. Men with lower absolute LQ, indicating mixed-hand use, were associated with significantly longer CAG-repeats. These data replicate previous findings by Hampson and Sankar in men (2012)^16^. Yet, the mechanism of X chromosome inactivation complicates statistical evaluation and interpretation of the data in women. At an early stage of human development, one of the two X chromosomes carried in each somatic cell is inactivated^36^. Consequently, from heterogeneous genotypes of the *AR* polymorphism firm conclusions cannot be drawn on the active *AR* allele. Yet, in homozygous genotypes the allelic expression is unambiguously the same, since inactivation of either allele will yield identical active alleles. In our study, 77 female participants were homozygous for the CAG-repeat expansion, and analyzing this subgroup separately revealed that longer CAG-repeats showed a tendency to be related with stronger left-handedness. These data support previous findings by Medland et al. (2005) who showed that left-handedness was more common among females with a greater number of CAG-repeats when considering the longer of the two alleles or the mean allele length of both alleles^11^. These results, therefore, suggest that handedness in both males and females is associated with the *AR* CAG-repeat length, with longer repeats being related to a higher incidence of non-right-handedness.

Interestingly, the lengths of the *AR* CAG-repeats have been demonstrated to be inversely related to the capacity of the receptor to activate target genes in vitro^37^,^38^,^39^,^40^. Within the normal range a 1.7% decrease in activity for each additional glutamine repeat could be demonstrated^31^. However, although the significant relation between weaker activity of AR with longer CAG-repeats has been demonstrated, the main determinant of phenotypic effects of this polymorphism is not completely understood. Recently, a large European study, comprising more than 2800 men found that longer CAG-repeats were associated with increased circulating levels of both testosterone and estradiol, indicating that the increased estrogen action and increased estrogen/androgen ratio in association with longer *AR* CAG-repeats could paradoxically play a more important role than the androgen-AR effect^33^.

Despite mechanistic differences (delayed left hemisphere development *vs.* asymmetric callosal axon pruning) the early theories developed by Geschwind and Witelson, both posit fetal testosterone as one common biological substrate that may be responsible for influencing sexually dimorphic patterns of brain development^18^,^20^. In non-human species, there is evidence for the organizational effects of pT on brain development that predict later expression of sex differences in brain and behavior^41^,^42^. However, human studies on this subject are much more complicated, and a major gap remains in understanding the mechanisms contributing to sex differences in the human brain.

The influence of fetal levels of testosterone was tested indirectly by comparing brain sizes of 9-year old dizygotic twins; children with a male *vs.* female co-twin had slightly larger brains^43^. Furthermore, it has been demonstrated that pT levels predicted differences in gray matter volume in some sexual dimorphic brain structures of boys aged 8–11^44^. Interestingly, gray matter density was recently found to be negatively related to testosterone levels, and this effect was more pronounced in those with shorter *AR* CAG-repeats with enhanced androgen sensitivity^45^. Shorter *AR* CAG-repeats were also found to be associated with a masculinization of the rate of cortical thickness change; in males greater androgen receptor efficiency was associated with a more masculine pattern of cortical maturation in bilateral regions of the inferior parietal lobule (IPL) known to underlie visuospatial tasks, whereas shorter repeats in females were associated with a more masculine pattern of cortical maturation in regions of the left inferior frontal gyrus (IFG), implicated in language and impulse-control domains, which favor females^46^.

Also the present results support a different mode of AR action in men and women, which may be explained by sex-specific feedback influence of AR on serum levels of androgens. Krithivas et al. suggest that longer *AR* CAG-repeats decrease AR activity in the hypothalamus resulting in decreased negative feedback and increased serum androgen levels in men^47^. In women, however, the hypothalamus-pituitary-gonadal axis is mainly regulated by female sex steroids. A study by Westberg et al. suggests that serum androgen levels in women are regulated by both the *AR* and *ER* beta genes^48^. Fewer *AR* CAG-repeats are associated with higher androgen levels in women. These data thus support the notion that differences in androgen levels as well as the *AR* CAG genotype in males as compared with females could contribute to between-sex differences in sexual dimorphisms in hemispheric asymmetry like handedness.

Altogether, our findings suggest that handedness in both males and females is associated with the length of the *AR* CAG-repeat. The observation that homozygous women with longer CAG-repeats were more often left-handed supports previous findings by Medland et al.^11^. Given that longer CAG-repeats in females, in contrast to males, are associated with lower testosterone levels, these results are in accordance with the callosal theory by Witelson that proposes that low prenatal testosterone levels result in less regressive development of temporo-parietal regions of the brain, resulting in a larger isthmus of the corpus callosum and less functional asymmetry, thus increasing left-handedness^19^,^20^,^48^. In line with this Grimshaw et al. (1995) found that female fetuses with lower levels of testosterone in the second trimester amniotic fluid were more likely to be left handed at age 10^49^. Regarding males, Hampson and Sankar (2012) also found that strong reliance on the left hand was associated with lower levels of testosterone, but no significant difference in *AR* repeat length was observed as compared to right-handers^16^. A significant association with longer CAG-repeats was, however, found with the group of mixed handed males, a fact that was confirmed in our results^16^. As already discussed by Hampson and Sankar, the main difference between the two groups of strict left- and mixed-hand preference and strict right-hand preference may be explained by differences in testosterone action in the developing brain, either mediated by differences in testosterone levels themselves or at the receptor level. However, finally both scenarios also correspond to the Witelson hypothesis.

The current study has some limitations. The classification system of handedness is controversial and tests available for assessing handedness are numerous and diverse in the way handedness is measured. Preference tests like the here used Edinburgh handedness inventory allow for handedness to be subjectively reported based on unimanual tasks. Yet, based on their high level of subjectivity and lack of consistent reliability the validity of the handedness report may be prone to errors^50^. Another limitation of our study is that we cannot completely exclude the possibility that the analyzed *AR* CAG-repeat may be in linkage disequilibrium with another functional variation.

In conclusion, our findings suggest that the length of the *AR* CAG-repeat could be one of the factors that lead to individual differences in hand preference perhaps through its influence on early developmental events that promote sexually dimorphic brain specialization. Providing insight into mechanisms that may give rise to the genetic and developmental basis of brain asymmetry shed light on cerebral specialization and also disorders that alter brain laterality.

## Methods

### Study participants

In total, 1057 genetically unrelated, healthy adult participants of Caucasian descent for at least two generations participated in the present study. One male participant had to be excluded from further analysis, because he did not fill out the handedness questionnaire, therefore 626 females and 430 males were included in the analysis. The participants had a mean age of 31.88 (SEM = 17.72; range: 18–93 years), and none of them had a history of any neurological or psychiatric diseases. No participants are included in the cohort that would have been forced to write with the right hand in school, although they have actually preferred the left. The study was approved by the ethics committee, Ruhr-University Bochum, Germany. All participants gave written informed consent and were treated in accordance with the declaration of Helsinki.

### Handedness assessment

Handedness was assessed using the Edinburgh handedness inventory^51^. In this questionnaire, participants have to indicate whether they prefer to use the left or right hand for ten different activities. Based on the participants answers, an individual laterality quotient (LQ) can be calculated using the formula LQ = [(R−L)/(R + L)] × 100 (R = number of right sided preferences; L = number of left-sided preferences). The LQ has a range between +100 and −100. Positive values indicate right handedness and negative values left handedness, while higher absolute values indicate more consistent handedness and lower absolute values more inconsistent handedness or ambidexterity. Based on each participant's individual LQ, three additional measures were calculated in order to separately investigate different aspects of handedness that may be associated with different genetic variations. In order to gain a measure of handedness direction (Direction 1), participants were grouped into right-handers (RH; LQ between 0 and 100) and left-handers (LH; LQ between−100 and 0), an approach that is commonly used in large-scale studies^6^. Additionally, we applied a different grouping method (Direction 2) used by Hampson and Sankar (2012) and grouped participants into right-handers (RH; LQ between 40 and 100); left-handers (LH; LQ between −100 and −40) and mixed handers (MH; LQ between −40 and 40). This was done the ensure comparability with the older study. Also, in order to gain a dichotomous measure of handedness consistency independent of the individuals preferred hand, participants were grouped into individuals with consistent (LQ either 100 or −100) or individuals with inconsistent (all other LQ's) handedness.

### Genotyping

For non-invasive sampling, exfoliated cells were brushed from the oral mucosa of the participants. DNA isolation was performed with QIAamp DNA mini Kit (Qiagen GmbH, Hilden, Germany). Genotyping of the AR exon 1 CAG repeat was performed by fragment analysis of the PCR product. The PCR products were electrophoresed on an ABI PRISM 3500xL Genetic Analyzer and the fragment lengths were determined using GeneMapper v4.1 Software. Oligonucleotides were designed using Primer Express 2.0 Software (Applied Biosystems). Further details of methodology and primer sequences are available upon request.

## Author Contributions

All authors contributed significantly to this work. L.A., S.O., S.S., V.N., W.M.G., J.T.E., O.G. and C.B. designed the research study, performed the research study and collected the data; J.G.H. and M.F. have provided a subset of samples, L.A. and S.O. analyzed the data and wrote the paper. All authors reviewed the manuscript. In addition, all authors approved the final draft.

## Supplementary Material

Supplementary Information Supplementary Figures

## Figures and Tables

**Table 1:** Handedness phenotype distributions for men, women and the overall sample

		Men (n = 430)	Women (n = 626)	Overall (n = 1056)
**LQ**	Mean	73.84	75.59	74.88
**Direction 1**	LH	8%	7%	8%
	RH	92%	93%	92%
**Direction 2**	LH	7%	6%	7%
	MH	3%	4%	3%
	RH	90%	90%	90%
**Consistency**	Inconsistent	53%	50%	51%
	Consistent	47%	50%	49%
