# Supplementary material for: Handedness and the X chromosome: The role of androgen receptor CAG-repeat length
Source: Sci Rep. 2015 Feb 9;5:8325. doi: 10.1038/srep08325 (PMC4321186; doi:10.1038/srep08325)
Supplement: Supplementary Information — Supplementary Figures [file srep08325-s1.pdf]

Supplementary figures

Handedness and the X chromosome: The role of androgen receptor CAG-repeat length

Larissa Arning*+[1], Sebastian Ocklenburg+[2], Stefanie Schulz[2], Vanessa Ness[2], Wanda M. Gerding[1], Jan G. Hengstler[3], Michael Falkenstein[3], Jörg T. Epplen[1], Onur Güntürkün[2], Christian Beste[4]

Affiliations

[1]Department of Human Genetics, Ruhr-University, 44780 Bochum, Germany

[2]Institute of Cognitive Neuroscience, Biopsychology, Department of Psychology, Ruhr-University, 44780 Bochum, Germany

[3]Leibniz Research Centre for Working Environment and Human Factors (IfADo), 44139 Dortmund, Germany

[4]Cognitive Neurophysiology, Department of Child and Adolescent Psychiatry, Faculty of Medicine of the TU Dresden, 01309 Dresden, Germany

Figure Captions for supplementary figures

Supplementary figure S1: LQ distribution for female and male participants.

Supplementary figure S2: Handedness Direction 1 phenotypes (left-handed, right-handed) in relation to CAG-repeat length for male (A) and female participants (B: short allele; C: long allele). Error bars show standard error.

Supplementary figure S3: Handedness Consistency phenotypes (consistent, inconsistent) in relation to CAG-repeat length for male (A) and female participants (B: short allele; C: long allele). Error bars show standard error.Figure Captions for supplementary figures

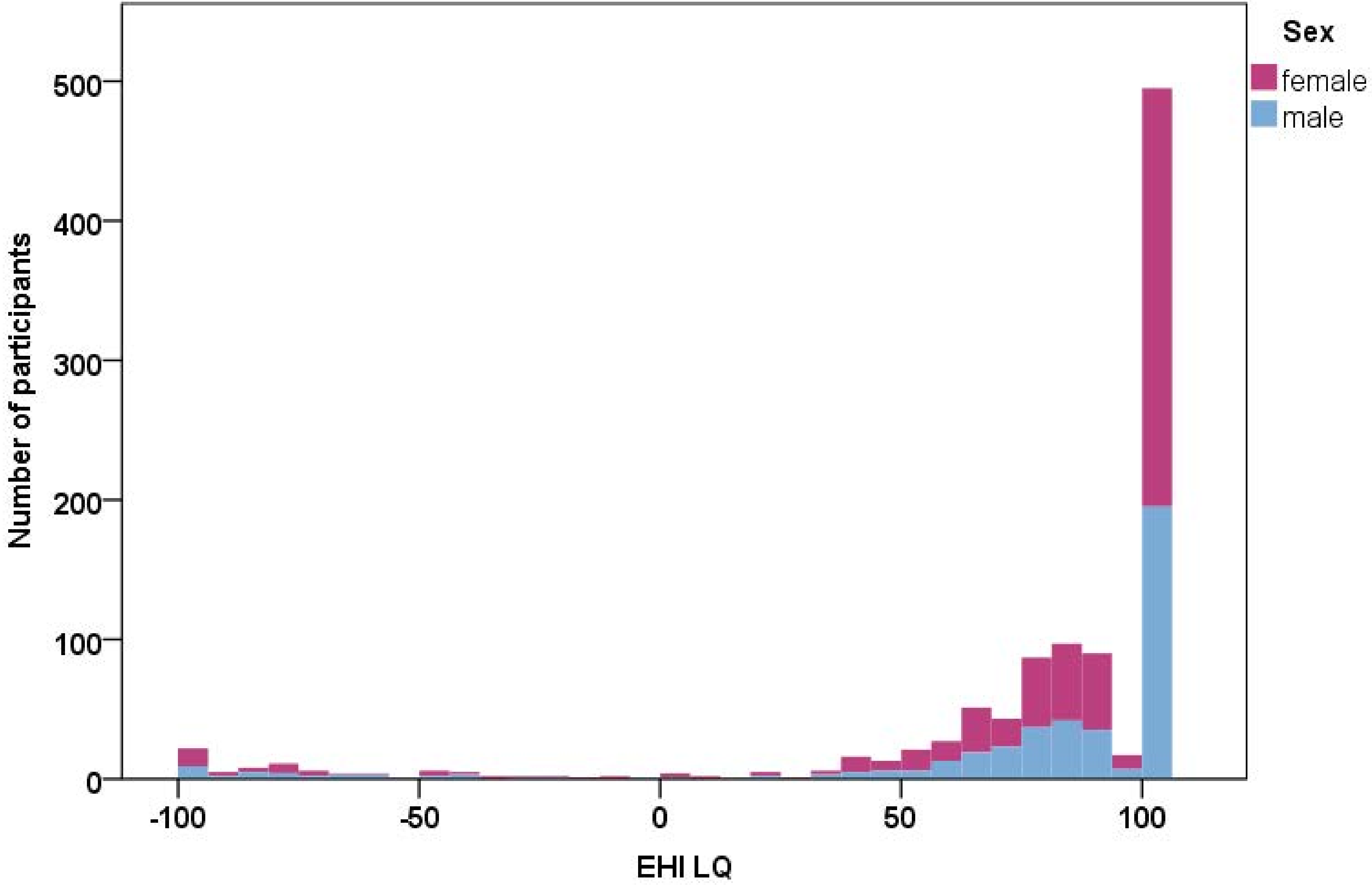

Sex
female
male
Number of participants
500
400
300
200
100
0
-100
-50
0
50
100
EHI LQ

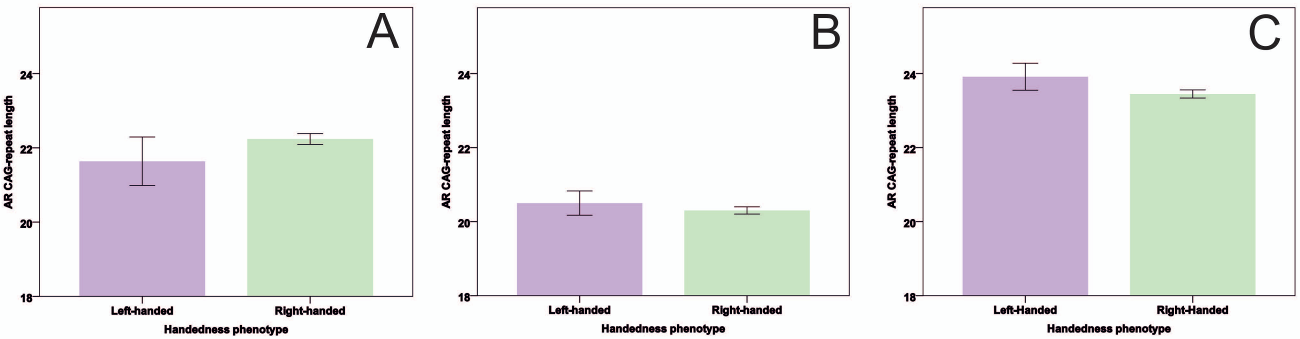

A
B
C
24
22
20
18
AR CAG-repeat length
Left-handed
Right-handed
Left-Handed
Right-Handed
Handedness phenotype

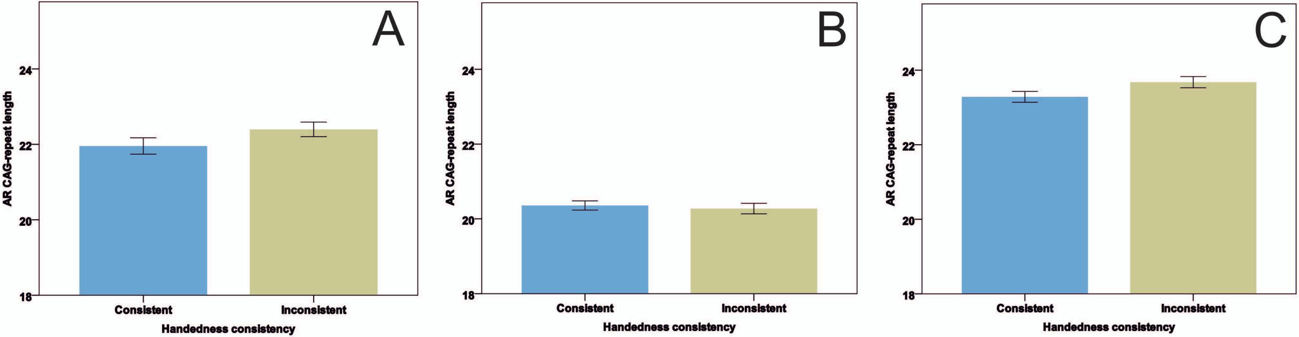
A
B
C
AR CAG-repeat length
24
22
20
18
Consistent
Inconsistent
Handedness consistency
